# Gut microbiota is correlated with gastrointestinal adverse events of metformin in patients with type 2 diabetes

**DOI:** 10.3389/fendo.2022.1044030

**Published:** 2022-11-17

**Authors:** Yuxin Huang, Xudan Lou, Cuiping Jiang, Xueying Ji, Xiaoming Tao, Jiao Sun, Zhijun Bao

**Affiliations:** ^1^ Department of Endocrinology, Huadong Hospital Affiliated to Fudan University, Shanghai, China; ^2^ Department of Gerontology, Huadong Hospital Affiliated to Fudan University, Shanghai, China

**Keywords:** type 2 diabetes, metformin, gastrointestinal adverse events, gut microbiota, short-chain fatty acids (SCFAs)

## Abstract

**Aim:**

Gastrointestinal discomfort is the most common adverse event in metformin treatment for type 2 diabetes. The mechanism of action of metformin is associated with gut microbiota. However, the gut microbial community structure related to metformin-induced gastrointestinal adverse events remains unclear. This study aimed to investigate it.

**Methods:**

50 patients with newly diagnosed diabetes were treated with metformin 1500mg/d for 12 weeks. The patients were divided into two groups according to whether gastrointestinal adverse events occurred (group B) or did not occur (group A) after treatment. The fecal bacterial communities and short-chain fatty acids (SCFAs) were sequenced and compared. 70 diabetes mice were randomly divided into 8 groups and treated with metformin (Met), clindamycin (Clin) and/or SCFA, which were the Met+/Clin+, Met+/Clin-, Met-/Clin+, Met-/Clin-, Met+/SCFA+, Met+/SCFA-, Met-/SCFA+ and Met-/SCFA- group. After 4 weeks of metformin treatment, blood glucose, food intake, fecal SCFAs, gut microbiota and gut hormones were measured.

**Results:**

Metformin increased the abundance of *Phascolarctobacterium*, *Intestinimonas* and *Clostridium III*. Functional prediction analysis showed that the propanoate metabolism pathway was significantly up-regulated. The concentrations of acetic acid and propanoic acid in feces were significantly increased. The abundance of *Clostridium sensu stricto, Streptococcus* and *Akkermansia* induced by metformin in group B was higher than that in group A. The propanoate metabolism pathway and propanoic acid in feces were significantly up-regulated in group B. In the animal experiments, the food intake decreased and glucose control increased in metformin groups compared with those in the control groups. The total GLP-1 level in the Met+/Clin- group was significantly higher than that in the Met-/Clin- group, while there was no statistical difference between the Met-/Clin- and Met+/Clin+ group. The total GLP-1 level in the Met-/SCFA+ group was significantly higher than that in the Met-/SCFA-group, while the levels of total GLP-1 and active GLP-1 in the Met+/SCFA- group and the Met+/SCFA+ group were significantly higher than those in the Met-/SCFA-group.

**Conclusions:**

Our data suggest that metformin promotes the secretion of intestinal hormones such as GLP-1 by increasing the abundance of SCFA-producing bacteria, which not only plays an anti-diabetic role, but also may causes gastrointestinal adverse events.

## Introduction

Type 2 diabetes is a metabolic syndrome that is primarily induced by β-cell dysfunction and insulin resistance (1). Insulin sensitivity and diabetes progression are closely related to the modulation of gut microbial composition (2). The insulin sensitizer metformin is recommended as the preferred, initial medication for the treatment of type 2 diabetes among numerous antidiabetic agents (3). It is effective and inexpensive, does not stimulate insulin secretion and does not cause hypoglycemia, and may reduce the risk of cardiovascular outcomes in diabetic people (4). However, the principal side effects of metformin are gastrointestinal adverse events due to abdominal pain, abdominal distention, diarrhea, nausea and inappetence. The high frequency (20-35%) of gastrointestinal adverse events was the most prevalent problem with metformin (5, 6), especially in elderly patients (about 54%) (7) and patients with Helicobacter pylori infection (about 62%) (8).

Two elegant studies by Forslund et al. in 2015 (6) and de la Cuesta-Zuluaga et al. in 2017 (9) addressed the drug signatures in the gut microbiome of diabetic patients. They indicated that metformin functioned by increasing short-chain fatty acids (SCFAs) production and elevating *Akkermansia* and *Escherichia*. The enteric nervous system may be directly or indirectly affected by the gut microbiota and its metabolites or signals (such as SCFAs, neurotransmitters, etc.), regulating the process of glucose and lipid metabolism in fat, liver, and brain. Both duodenal total glucagon-like peptide-1 receptor (GLP-1R)-protein kinase A signaling and a neuronal-mediated gut-brain-liver pathway were required for metformin to lower hepatic glucose production and plasma glucose levels (10). However, the mechanism of gastrointestinal adverse effects of metformin remains not fully understood until now. Metformin is highly water-soluble, which can irritate the gastrointestinal mucosa after entering the gastrointestinal tract. In addition, metformin increases the bile acid pool within the intestine predominantly through reduced ileal absorption (11). This disruption of the enterohepatic circulation of bile salts has potential consequences for diarrhea. The alteration in bile acid absorption may increasing the concentration of intestinal peptide GLP-1 and causing upper digestive tract discomfort (12).

Our hypothesis suggests that metformin may cause gastrointestinal adverse events by regulating gut microbiota. This study aimed to illustrate the gut microbial community structure underlying metformin-induced gastrointestinal adverse events in elderly patients with type 2 diabetes and in diabetic mice. The difference in gut microbial community structure between diabetic patients with and without gastrointestinal adverse events was compared and illustrated. Also, the bacteria and clinical factors that might be associated with the incidence of metformin-induced adverse events were identified. Furthermore, we aimed to explore the relationship between gut microbiota, SCFAs, gut hormones and metformin-induced gastrointestinal adverse events in animal experiments.

## Material and methods

### Patients enrollment and study protocol

50 antidiabetic agents treatment-naïve patients with newly diagnosed type 2 diabetes, aged≥60 years, with body mass index≥18.5 kg/m^2^ and Hemoglobin A1c (HbA1c) of 7.0%-9.0% were enrolled from a previous study (7). The exclusion criteria included the following: (a) confirmed or suspected type 1 diabetes; (b) previous treatment with insulin or other antidiabetic drugs for more than 14 days; (c) a history of known peptic ulcers, Helicobacter pylori infection, gastrointestinal surgery, chronic gastritis, gastrointestinal tumor or severe gastrointestinal discomfort; (d) current (within 3 months of screening) diabetic ketoacidosis or hyperosmolar coma; (e) current cardiovascular disease or other serious disease; (f) a creatinine clearance rate <60 mL/min; (g) liver enzymes more than 2 times the upper limit of normal at screening; (h) use of unknown combination drugs; and (i) poor drug compliance. This study was approved by the Ethics Committee of Huadong Hospital Affiliated to Fudan University, Shanghai, China (Ethics Number: 2018K065). Written informed consent was obtained from all subjects prior to the study.

**Figure 1:**
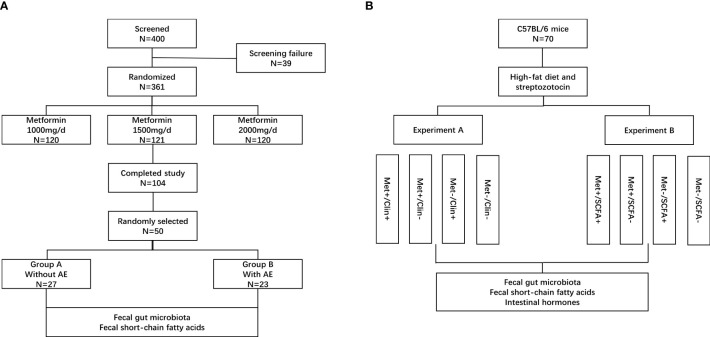
Patient disposition and study protocol. AE, adverse events; Met, metformin; Clin, clindamycin; SCFA, short-chain fatty acid. **(A)** clinical trial; **(B)** animal experiments.

Eligible people were randomized 1:1:1 to receive 1000 mg/d, 1500 mg/d or 2000 mg/d of metformin (Sino-American Shanghai Squibb Pharmaceuticals Ltd., Shanghai, China). This study has a 12 weeks treatment period. Biochemical measurements of plasma glucose, insulin and HbA1c were performed in a central laboratory at baseline and after 12 weeks. Body mass index (BMI, kg/m^2^) was calculated by dividing body weight by the square of height. HbA1c was determined by high pressure liquid phase method (Bio-Rad variant II, USA), insulin was detected by chemiluminescence (Snibe MAGLUMI 4000, China); glucose was measured by hexokinase Colorimetry (HITACHI 7600 Series, Japan). Insulin sensitivity was calculated as the homeostasis model assessment of insulin resistance index (HOMA-IR) by using the HOMA Calculator (Headington, Oxford, UK; http://www.dtu.ox.ac.uk). The fecal samples were randomly collected from 50 patients in the metformin 1500m/d group before and after treatment. The patients were divided into two groups according to whether gastrointestinal adverse events occurred (group B) or did not occur (group A) after treatment. The fecal bacterial communities and SCFAs were tested and compared (**Figure 1A**). The SCFAs (including acetic acid, propanoic acid and butyric acid) in feces were determined by gas chromatography/mass spectrometry. Fecal samples were homogenized and diluted with distilled-deionized water in a ratio 1:1. An aliquot of 1 g was spiked with a combined standard solution of SCFAs diluted in water (organic acid kit ref. 47264, Supelco, Bellefonte, PA) to obtain curves in the range 25–750 ng/mL. The analytes were injected in the splitless mode into a gas chromatography system (Agilent GC6890, USA). The chromatographic peaks were checked for homogeneity using the extracted ions of the characteristic fragments to optimize the resolution and peak symmetry (13).

### DNA extraction, gut microbiota analysis and data processing

Three DNA samples were extracted from each fecal sample collected from patients in the group A (n=27) and the group B (n=23) before and after treatment using the QIAamp DNA stool mini kit (Qiagen, Hilden, Germany). DNA samples at a dilution of 1 ng/μL were used as templates for amplification targeting the V4 variable region of the 16S rRNA with the barcoded primers (515F/806R). The high-fidelity DNA polymerase (New England Biolabs, Ipswich, MA, USA) and Phusion^®^ High-Fidelity PCR Master Mix kit (New England Biolabs) was used for the amplification. PCR products were used for the construction of the DNA libraries using the TruSeq^®^ DNA PCR-Free sample preparation kit (Illumina Inc., San Diego, CA, USA). The 16s rDNA sequencing was performed on the Illumina MiSeq platform (Illumina Inc., San Diego, CA, USA). The sequencing data in the format of FASTQ was processed using the FLASH software (14). After data filtering using the Usearch and Uchime software, raw tags were quality-filtered using the Qiime software (15). The clustering of operational taxonomic units (OTU) was performed using the Usearch software (16). Bacterial taxonomies at the levels of phylum, family, and genus were annotated using the RDP classifier based on the Bergey’s taxonomy and Naïve Bayesian assignment. The ribosomal RNA sequences were annotated in the SILVA ribosomal RNA gene database based on the same criteria. The alpha diversity indexes were analyzed using the Qiime (15) and the beta-diversity metrics were calculated using the weighted UniFrac algorithm (Vegan package R, version 2.0-10). Principal coordinate analysis (PCoA) was performed for all samples. The iconic bacteria in each group were identified using the linear discriminant analysis (LEfSe, version 1.1.0). The statistical significance among groups was assessed using the analysis of similarities (ANOSIM) of weighted UniFrac distances. The functional profiles of prokaryotic communities were predicted using Tax4Fun2 based on 16S rRNA sequencing data (17).

### Animal experiments

The animal study was reviewed and approved by the Ethics Committee of Fudan University (Ethics Number: 202101004S). A total of 70 male 8-week-old C57BL/6 mice [Beijing Vital River Laboratory Animal Technology Co., Ltd., China, animal production license number: SCXK(Beijing) 2021-0011] were selected and treated with high-fat diet (Trophic Animal Feed High-Tech Co., Ltd., China) combined with low dose of streptozotocin (Merck KGaA, Darmstadt, Germany) to establish a type 2 diabetic mouse model. After four weeks on the high fat diet, the mice were treated with 75 mg/kg streptozotocin followed three days later with a second dose of streptozotocin (50 mg/kg) as needed. Mice with blood glucose ≥13.8 mmol/L were considered diabetic (18). It has been reported that intake of clindamycin reduced the total concentration of SCFAs in faeces (19). The mice were randomly divided into two experiments and treated with metformin (Sino-American Shanghai Squibb Pharmaceuticals Ltd., Shanghai, China, 200mg/kg/d), clindamycin (Shandong Fangming Co., Ltd., China, 15mg/kg/d) and/or propanoate (MedChemExpress LLC., USA, 300mg/kg/d). There were four groups in experiment A: 9 mice in the metformin and clindamycin group (Met+/Clin+), 8 mice in the metformin group (Met+/Clin-), 9 mice in the clindamycin group (Met-/Clin+), and 8 mice in the control group (Met-/Clin-). There were four groups in experiment B: 9 mice in the metformin and propanoate group (Met+/SCFA+), 9 mice in the metformin group (Met+/SCFA-), 9 mice in the propanoate group (Met-/SCFA+), and 9 mice in the control group (Met-/SCFA-) (**Figure 1B**).

After 4 weeks of metformin treatment, the body weight, blood glucose, food intake, intestinal hormones, fecal SCFAs and gut microbiota were measured. Glucose was measured by portable blood glucose meter (Roche ACCU-CHEK Performa). Intestinal tissue of mice was immersed in phosphate buffer solution (PBS). Dipeptidyl peptidase-4 (DPP-IV) inhibitor was rapidly added. Then it was homogenized, centrifuged and diluted to an appropriate concentration. The levels of total glucagon-like peptide-1 (GLP-1), active GLP-1 and peptide YY (PYY) in intestinal tissue were determined by ELISA according to the kit instructions (Millipore ELISA kits, USA) (20). The expression levels of GLP-1, GLP-1 receptor (GLP-1R) and PYY in intestinal tissue were detected by immunofluorescence. The intestinal tissues were paraffin embedded and sectioned. After baking overnight in the oven, xylene dewaxing was performed the next day. Then it was soaked with gradient alcohol (100%, 95%, 70%, 50%). They were incubated with primary antibody to GLP-1, GLP-1R and PYY (abcam, UK) at 4° C overnight. Sections were stained by 4’,6-diamidino-2-phenylindole (DAPI, abcam, UK) and washed for 3 times with PBS. GLP-1, GLP-1R, PYY and DAPI were observed by a fluorescence microscope (Olympus, Japan) after triggered at 594 nm and 358 nm respectively (21). Image was analyzed quantitatively by Image J 1.8.0 (National Institutes of Health, USA). The SCFAs (including acetic acid, propanoic acid and butyric acid) in feces were determined by gas chromatography/mass spectrometry and according to the method described by de la Cuesta-Zuluaga et al. (13). Fecal bacterial communities in experiment A were determined using the method described above.

### Statistical analyses

The demographic data was analyzed using the SPSS 23.0 software (SPSS Inc, Chicago, USA). Based on the result of the Kolmogorov-Smirnov test, normally and non-normally distributed continuous variables were expressed as mean ± standard deviation and median (interquartile range), respectively. The differences in dichotomous variables between groups were analyzed using the χ2 test, and the differences in continuous variables between groups were analyzed using the Mann-Whitney U test or t-test. Logistic regression analysis was performed to identify the bacteria or factors associated with metformin-induced gastrointestinal adverse effects and to calculate the 95% confident interval (CI) and odds ratio (OR). For all analyses, P values < 0.05 were considered statistically significant. The statistical diagrams were plotted using GraphPad Prism 5 (Graphpad software, Inc., La Jolla, CA, USA).

## Results

### Characteristics of patients

A total of 50 patients (27 males and 23 females) were included according to the inclusion and exclusion criteria. The average age of all patients was 68.52 ± 6.45 years and the average baseline fasting glucose was 8.13 ± 1.89 mmol/L. 23 patients (46.00%) developed gastrointestinal adverse events after metformin treatment. Accordingly, all patients were assigned into two groups: patients with adverse events (n = 23) in group B and patients without adverse events (n = 27) in group A. The baseline characteristics of patients assigned to the two groups are shown in **Table 1**. Patients with metformin-induced adverse events had a lower body height (161.61 ± 8.94 cm) compared with patients without adverse events (167.50 ± 8.22 cm; P= 0.019). There was no difference in the other variables between patients with and without adverse events, including the age, gender, body mass index, blood pressure, fasting glucose (before and after treatment), HbA1c (before and after treatment), HbA1c reduction, and fecal SCFAs (before treatment, including acetic acid, propanoic acid, butyric acid). After metformin treatment for 12 weeks, the concentrations of acetic acid and propanoic acid were significantly increased in both group A and group B (all *P*<0.05). The concentration of propanoic acid in feces in group B was significantly higher than that in group A after treatment (P<0.05).

**Table 1:** Characteristics of patients with type 2 diabetes.

Variables	Group A (without AE, n=27)	Group B (with AE, n=23)	*P* value
Age (years)	68.20 ± 7.03	68.74 ± 5.77	0.436^a^
Height (cm)	167.50 ± 8.22	161.61 ± 8.94	***0.019^a^***
Male/female	18/9	9/14	0.087^b^
Body mass index (kg/m^2^)	24.79 ± 2.89	25.82 ± 2.88	0.217 ^a^
Systolic BP (mmHg)	134.00 ± 10.83	133.78 ± 16.01	0.470 ^a^
Diastolic BP (mmHg)	74.82 ± 6.72	77.70 ± 8.58	0.189 ^a^
FBG (mmol/L; before T)	7.96 ± 1.91	8.20 ± 1.93	0.225 ^a^
FBG (mmol/L; post T)	7.52 ± 1.46	7.71 ± 1.71	0.267 ^a^
Fasting insulin (mIU/L)	9.02 ± 4.67	8.89 ± 4.54	0.932 ^a^
HOMA-IR (before T)	3.23 ± 1.89	3.22 ± 2.01	0.798^a^
HbA1c (%; before T)	8.09 ± 0.47	8.1 ± 0.5	0.934 ^a^
HbA1c (%; post T)	7.22 ± 0.62	7.16 ± 0.56	0.840 ^a^
HbA1c reduction	1.03 ± 0.29	0.93 ± 0.44	0.218 ^a^
Acetic acid (mmol/L, before T)	39.9 ± 4.3	38.0 ± 5.1	0.552 ^a^
Propanoic (mmol/L, before T)	2.6 ± 0.7	2.4 ± 0.8	0.347 ^a^
Butyric (mmol/L, before T)	1.5 ± 0.5	1.7 ± 0.4	0.377 ^a^
Acetic acid (mmol/L, post T)	45.6 ± 7.3	44.2 ± 8.1	0.533 ^a^
Propanoic (mmol/L, post T)	3.0 ± 0.8	3.5 ± 0.7	***0.022 ^a^***
Butyric (mmol/L, post T)	1.6 ± 0.5	1.9 ± 0.7	0.265 ^a^

a and b, statistical analysis by t-test and χ^2^ test, respectively.

Normally distributed data were expressed as mean ± standard deviation.

AE, adverse events; FBG, fasting blood glucose; T, treatment;

HbA1c, hemoglobin Alc; HOMA-IR, homeostasis model assessment of insulin resistance.

### Summary of the illumina sequencing data

**Figure 2:**
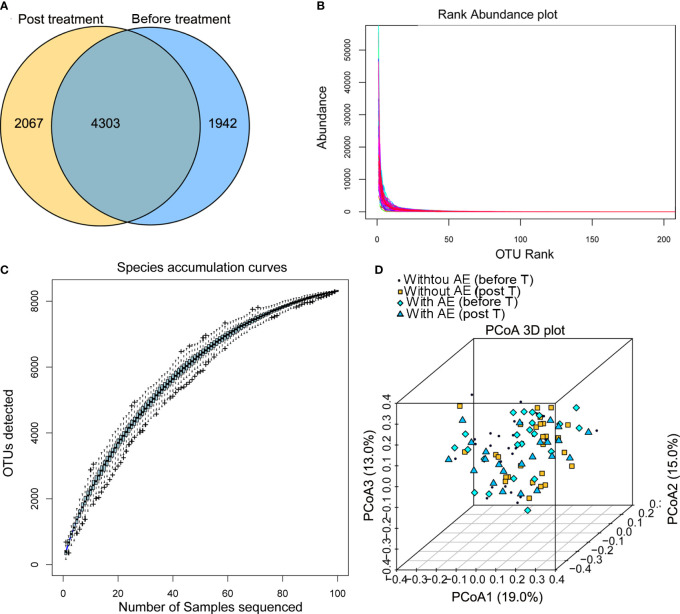
Summary of the Illumina sequencing data. **(A)** OTUs of samples collected from patients before and after treatment; **(B)** the sample rank-abundance curve; **(C)** species accumulation curve; **(D)** the PCoA analysis. AE, adverse events; T, treatment.

After Illumina sequencing and data processing, 53,968 - 95,508 clean reads were obtained in each sample. After OTU annotation, 8,312 OTUs were identified, including 4,303 OTUs common to the stool samples collected from patients before and after treatment (**Figure 2A**). There was no difference in the alpha diversity indexes (ACE, Shannon, Chao1, and Simpson) between samples collected before and after treatment or among samples collected from patients with and without adverse events. The fact that the sample rank-abundance curve (**Figure 2B**) and species accumulation curve (**Figure 2C**) reached a plateau showed deeper sequencing will not increase bacterial diversity. Also, the PCoA analysis based on the weighted UniFrac distances and Bray-Curtis dissimilarity of OTUs showed that there was no distinct group boundary among the samples sequenced (**Figure 2D**).

### Metformin-induced differences in bacteria composition

**Figure 3:**
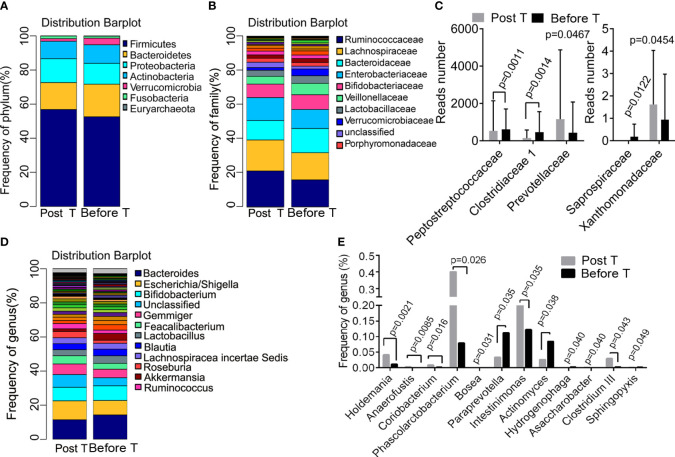
Metformin-induced differences in bacteria composition. **(A)** the dominant phyla before and after treatment; **(B)** the dominant family before and after treatment; **(C)** the different family before and after treatment; **(D)** the dominant genera before and after treatment; **(E)** the different genera before and after treatment. T, treatment.

The differences in the abundance of OTUs at different levels were compared between samples collected from patients before and after metformin treatment. Taxonomy annotation showed that the dominant phyla were *Firmicutes* (52.24% and 56.46%), *Bacteroidetes* (19.00% and 15.80%), *Proteobacteria* (12.37% and 14.42%), and *Actinobacteria* (11.04% and 9.94%; **Figure 3A**) in diabetic patients. At the family level, *Ruminococcaceae* (20.99% and 15.80%), *Lachnospiraceae* (18.17% and 15.89%), *Bacteroidaceae* (11.38% and 14.21%), *Enterobacteriaceae* (13.48% and 11.14%), and *Bifidobacteriaceae* (7.92% and 8.63%; **Figure 3B**) had a relatively high abundance before and after metformin treatment. We found that 12-week metformin treatment increased the abundance of the family *Prevotellaceae* (P=0.0467) and *Xanthomonadaceae* (P=0.0454), but decreased the abundance of the family *Peptostreptococcaceae* (P=0.0011), *Clostridiaceae* 1 (P = 0.0014), and *Saprospiraceae* (P=0.0122) significantly in patients (**Figure 3C**). Besides, the dominant genera in diabetic patients were *Bacteroides* (11.38% and 14.21%), *Escherichia/Shigella* (11.08% and 8.47%), and *Bifidobacterium* (7.92% and 8.61%; **Figure 3D**). Metformin treatment induced significantly differences in the abundance of 12 genera, including *Holdemania* (P=0.0021), *Anaerofustis* (P=0.0085), *Coriobacterium* (P=0.016), *Phascolarctobacterium* (P=0.026), *Actinomyces* (P=0.038), *Clostridium III* (P=0.049), *Intestinimonas* (P=0.035) and *Paraprevotella* (P=0.035, **Figure 3E**). Functional prediction analysis showed that pathways such as the pantothenate and CoA biosynthesis, protein export, necroptosis, propanoate metabolism were significantly up-regulated after metformin treatment.

### Differences in bacterial abundance between patients with and without adverse events

**Figure 4:**
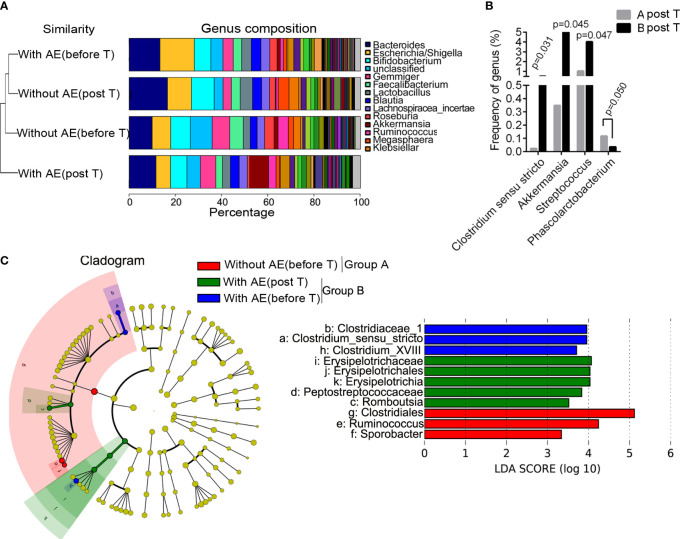
Differences in bacterial abundance between patients with and without adverse events. **(A)** the group similarity of samples at the genus level; **(B)** the different genera in patients without and with gastrointestinal adverse events after treatment; **(C)** the LEfSe analysis. AE, adverse events; T, treatment.

**Figure 4A** shows the group similarity of samples at the genus level. There was not significant distance in the genus composition between patients with and without adverse events before treatment. We identified that the abundance of five genera were significantly different in patients without and with gastrointestinal adverse events after treatment, including *Clostridium sensu stricto* (P=0.031), *Akkermansia* (P=0.045), *Streptococcus* (P=0.047), *Rhizobium* (P=0.019) and *Phascolarctobacterium* (P =0.050; **Figure 4B**). The STAMP analysis showed that the abundance of *Clostridium sensu stricto* (P=0.031), *Akkermansia* (P=0.046) and *Streptococcus* (P=0.047) was significantly higher in group B than that in group A after treatment. Besides, the LEfSe analysis showed that *Romboutsia, Erysipelotrichaceae, Erysipelotrichales, Erysipelotrichia*, and *Peptostreptococcaceae* were dominant bacteria in patients with metformin-induced gastrointestinal adverse events (**Figure 4C**). Functional prediction analysis showed that pathways such as pentose and glucuronate interconversions, pantothenate and CoA biosynthesis, biosynthesis of secondary metabolites, phenylalanine, tyrosine and tryptophan biosynthesis, aminoacyl-tRNA biosynthesis, biofilm formation of *Escherichia coli*, biosynthesis of amino acids, propanoate metabolism in feces were significantly up-regulated in patients with adverse events after metformin treatment.

The results of logistic regression analysis showed that body height was a protective factor against metformin-related gastrointestinal adverse events (OR=0.913, 95%CI 0.844-0.986, P=0.021). *Akkermansia* (OR=1.045, 95%CI 1.007-1.112, P=0.032) and *Streptococcus* (OR=1.121, 95%CI 1.033-1.262, P=0.046) were risk factors for metformin-related upper gastrointestinal adverse events (including abdominal pain, nausea, vomiting, bloating and anorexia).

### Animal experiment A

**Figure 5:**
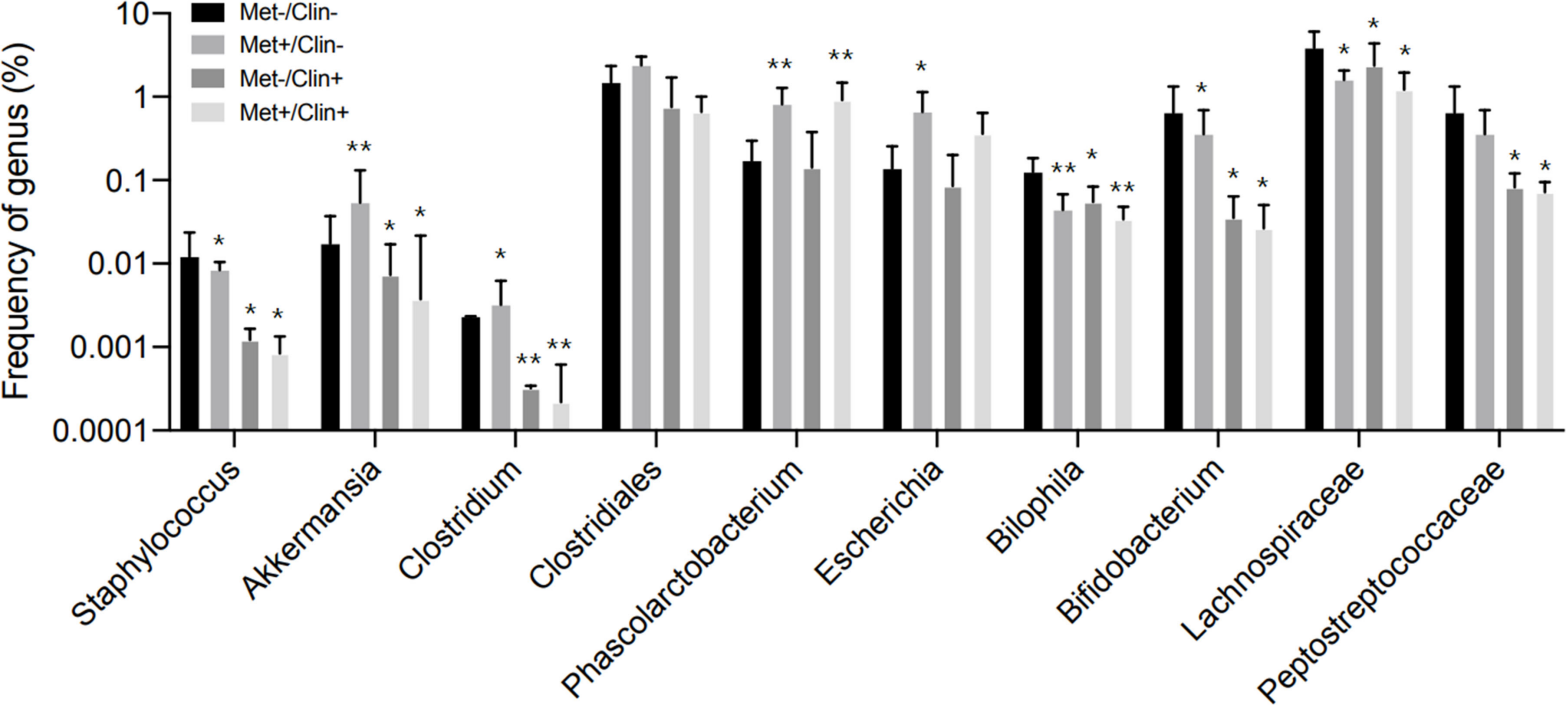
Frequency of genus in experiment A. Met, metformin; Clin, clindamycin. *Compared with the Met-/Clin- group, *P*<0.05; **Compared with the Met-/Clin- group, *P*<0.01.

After four weeks of treatment, the food intake of mice in the Met-/Clin+ group, the Met+/Clin- or the Met+/Clin+ group decreased compared with that in the Met-/Clin- group (P<0.05). The fasting glucose and 2h glucose of mice in the Met+/Clin- group and the Met+/Clin+ group were lower than those in the Met-/Clin- group (P<0.05). The levels of fecal acetic acid and propanoic acid of mice in the Met-/Clin+ and the Met+/Clin+ group were significantly decreased (P<0.01), while the fecal propanoic acid and butyric acid in the Met+/Clin- group were significantly increased (P<0.05). The total GLP-1 level in the Met+/Clin- group was significantly higher than that in the Met-/Clin- group (P<0.01), and there was no statistical difference in the levels of the total GLP-1, active GLP-1 and PYY of mice between the Met-/Clin- group and the Met+/Clin+ group (P>0.05; **Table 2A**). The abundance of *Akkemansia* (P<0.01), *Clostridium* (P<0.05), *Phascolarctobacterium* (P<0.01) and *Escherichia* (P<0.05) in the Met+/Clin- group increased significantly, while the abundance of *Staphylococcus* (P<0.05), *Akkemansia* (P<0.05), *Clostridium* (P<0.01), *Bilophila* (P<0.05), *Bifidobacterium* (P<0.05), *Lachnospiraceae* (P<0.05) and *Peptostreptococcaceae* (P<0.05) in the Met-/Clin+ group and the Met+/Clin+ group decreased significantly, compared with those in the Met-/Clin- group (**Figure 5**).

**Table 2A:** Characteristics of mice in experiment A (after treatment).

Variables	Met-/Clin-	Met-/Clin+	Met+/Clin-	Met+/Clin+
Food intake (g/d)	4.3 ± 0.6	3.6 ± 0.6*	3.6 ± 0.5*	3.5 ± 0.4*
Weight (g)	42.1 ± 2.8	41.4 ± 3.3	42.5 ± 4.6	40.8 ± 5.0
Fasting glucose (mmol/L)	25.0 ± 5.7	25.2 ± 4.5	16.4 ± 4.4**	16.2 ± 5.2**
2h glucose (mmol/L)	30.4 ± 6.2	30.8 ± 7.0	20.4 ± 5.6**	21.8 ± 7.3**
Acetic acid (mmol/L)	35.6 ± 3.3	23.6 ± 4.5**	35.1 ± 2.5	26.7 ± 3.7**
Propanoic acid (mmol/L)	14.7 ± 1.5	10.0 ± 1.5**	16.9 ± 1.7*	9.6 ± 1.9**
Butyric acid (mmol/L)	3.9 ± 0.8	3.3 ± 0.8	5.9 ± 0.8**	4.5 ± 1.0
Total GLP-1 (pmol/L)	28.4 ± 4.4	30.2 ± 4.2	37.4 ± 6.3**	29.2 ± 7.0
Active GLP-1 (pmol/L)	3.5 ± 0.5	3.3 ± 0.6	3.7 ± 0.6	3.6 ± 0.7
PYY (pmol/L)	12.0 ± 2.1	10.8 ± 1.1	10.3 ± 1.4	11.8 ± 3.1

*Compared with the Met-/Clin- group, P<0.05; **Compared with the Met-/Clin- group, P<0.01.

### Animal experiment B

**Figure 6:**
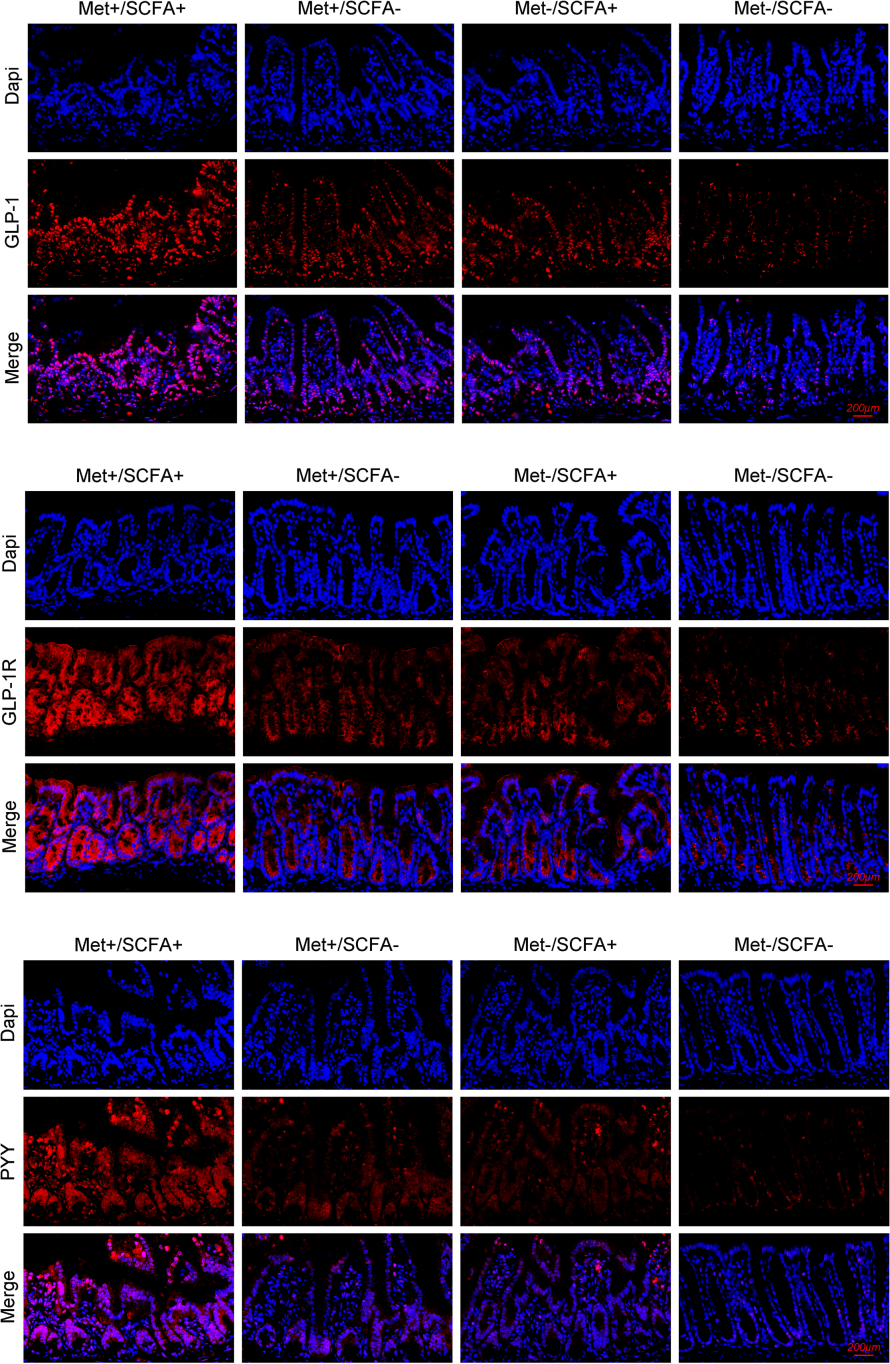
The expression levels of GLP-1, GLP-1R and PYY. Met, metformin; Clin, clindamycin; SCFA, short-chain fatty acid.

After four weeks of treatment, the food intake of mice in the Met-/SCFA+ group and the Met+/SCFA- group decreased compared with that in the Met-/SCFA- group (P<0.05), while the food intake in the Met+/SCFA+ group decreased more significantly (P<0.01). The fasting glucose and 2h glucose of mice in the Met+/SCFA- group and the Met+/SCFA+ group were lower than those in the Met-/SCFA- group (P<0.05). The levels of fecal propanoic acid of mice in the Met-/SCFA+ group, the Met+/SCFA- group and the Met+/SCFA+ group were significantly higher than those in the Met-/SCFA- group (P<0.05). The total GLP-1 level in the Met-/SCFA+ group (P<0.05), and the levels of total GLP-1 and active GLP-1 in the Met+/SCFA- group and the Met+/SCFA+ group were significantly higher (P<0.05; **Table 2B**) than those in the Met-/SCFA- group. The expression levels of GLP-1 in the Met+/SCFA- group and the Met-/SCFA+ group (P<0.05), and GLP-1, GLP-1R and PYY in the Met+/SCFA+ group increased significantly (P<0.01; **Figure 6**) compared with those in the Met-/SCFA- group.

**Table 2B:** Characteristics of mice in experiment B (after treatment).

Variables	Met-/SCFA-	Met-/SCFA+	Met+/SCFA-	Met+/SCFA+
Food intake (g/d)	4.5 ± 0.4	3.8 ± 0.6*	3.9 ± 0.6*	3.5 ± 0.6**
Weight (g)	43.5 ± 3.8	42.0 ± 5.3	44.5 ± 5.7	40.7 ± 5.6
Fasting glucose (mmol/L)	24.2 ± 5.0	24.2 ± 4.5	17.5 ± 4.7**	18.8 ± 5.2*
2h glucose (mmol/L)	29.3 ± 5.2	30.7 ± 4.0	20.6 ± 3.9**	21.2 ± 5.0**
Acetic acid (mmol/L)	35.9 ± 3.0	35.0 ± 3.1	36.0 ± 2.9	36.8 ± 3.7
Propanoic acid (mmol/L)	15.4 ± 2.0	19.4 ± 1.8**	17.0 ± 1.6*	19.1 ± 1.7**
Butyric acid (mmol/L)	4.5 ± 0.7	4.3 ± 0.4	5.9 ± 0.7**	5.3 ± 0.8*
Total GLP-1 (pmol/L)	29.9 ± 5.0	35.3 ± 3.4*	44.5 ± 5.9**	49.8 ± 10.1**
Active GLP-1 (pmol/L)	3.3 ± 0.6	3.5 ± 0.5	4.1 ± 0.7*	4.9 ± 0.9**
PYY (pmol/L)	13.4 ± 1.8	12.1 ± 1.9	13.2 ± 1.7	15.0 ± 2.5

*Compared with the Met-/SCFA- group, P<0.05; **Compared with the Met-/SCFA- group, P<0.01.

## Discussion

The gut microbiome has prevalent roles in human health through modulating intestinal ecology and physiology and the metabolic and immune system of the host (22–24). Hypoglycemic drugs like α-glucosidase inhibitors, DPP-4i, and metformin influence the structure of the human gut microbial community (6, 25). Previous studies showed that the abundance of *Ruminococcus*, *Phascolarctobacterium*, *Intestinimonas*, *Corynebacterium*, *Megasphaera*, and *Akkermansia* were changed by metformin in animal models and humans (6, 26–29). These results confirmed the influence of metformin treatment on the structure of gut microbial community in diabetic patients.

Metformin is an old drug with a lot of history and still much more to tell us. With the advance in clinical and experimental science, always newer pleiotropic effects come out, making it one of the most valuable drugs available. Metformin has good effects on lowering glucose, improving insulin sensitivity and lipid profile, reducing cardiovascular events and mortality, and preventing progression to heart failure in patients with diabetes (30, 31). Endothelial dysfunction is a well-known important risk factor for the development of diabetes cardiovascular complications. Adenosine 5’-monophosphate-activated protein kinase pharmacological activation plays a key role, with metformin inhibiting inflammation and improving endothelial dysfunction. The effects of metformin on endothelial dysfunction seem to be among the main factors responsible for the cardiovascular prevention (32). Metformin also displays significant growth inhibitory and proapoptotic effects in several cancer models. More preclinical data support the role of metformin as an adjuvant drug in the treatment of lung cancer (33). Metformin can decrease insulin resistance, increase the utilization of glucose and the production of lactic acid, and promote the secretion of postprandial GLP-1 in the intestine (34–36). A previous report has shown that the SCFA-producing bacteria decrease in patients with diabetes and increase after metformin treatment (37). Bacterial SCFAs influence innate and adaptive immunity of the host and play an anti-inflammatory role (38). The decreased abundance of SCFA-producing bacteria in fecal has been associated with an increased incidence of diabetes (37, 38). The metformin treatment for diabetic animals and patients significantly increased the abundance of *Akkermansia* (9), and other SCFA-producing bacteria like *Ruminococcus*, *Phascolarctobacterium*, and *Intestinimonas* (39, 40). It has been reported that the increased abundance of SCFA-producing bacteria was associated with improved insulin sensitivity (39). Therefore, even the increased prevalence in gastrointestinal adverse events, which are surely detrimental for patients’ quality of life and adherence, metformin should be provided as the first-choice drug and changed only in case of intolerance (41, 42).

Our present study demonstrated that the 12-week metformin treatment decreased the abundance of *Actinomyces* and *Paraprevotella*, but increased the abundance of SCFA-producing gut bacteria, including *Phascolarctobacterium*, *Clostridium III*, and *Intestinimonas*. Functional prediction analysis showed that pathways such as propanoate metabolism were significantly up-regulated after metformin treatment. The concentrations of acetic acid and propanoic acid increased significantly after metformin treatment. However, the concentrations of acetic acid did not increase in diabetic mice after metformin treatment. This may be due to the species difference between human and mice. These results showed that metformin-mediated glucose control in diabetic patients were mediated by modulating the composition of SCFA-producing bacteria.

We further analyzed and compared the gut microbiota between diabetic patients with and without gastrointestinal adverse events after taking metformin. Comparison analysis indicated that metformin also increased the abundance of *Clostridium sensu stricto*, *Akkermansia* and *Streptococcus* in patients with gastrointestinal adverse events compared with that in patients without adverse events. *Akkermansia* and *Streptococcus* were both risk factors for metformin-related upper gastrointestinal adverse events in patients aged > 60 years old. Propanoate metabolism were significantly up-regulated and the concentration of propanoic acid in feces was significantly higher in patients with gastrointestinal adverse events after treatment. These results are somewhat regrettable because the increase in the abundance of SCFA-producing bacteria is very important in the hypoglycemic mechanism of metformin. However, our results showed that the increased abundance of some SCFA-producing bacteria might also be related to the gastrointestinal adverse effects of metformin.

Interestingly, here we for the first time showed that body height might be a protective factor against metformin-induced gastrointestinal adverse effects, as patients (>60 years old) without adverse events had a higher body height compared with patients with adverse events. Lower body height correlates with a high risk of diabetes, especially in women (43, 44). Higher height means larger body surface area. A previous study presented that metformin AUC_0-48h_ was inversely associated with body surface area (45). This present study showed that diabetic patients (>60 years old) who had a relatively high body height might be at a low risk of gastrointestinal adverse events following the metformin treatment.

The mechanism by which gastrointestinal adverse events are induced by metformin is not fully understood. The reported gastrointestinal adverse events may be caused by bile salt reabsorption (46), gut microbiota alteration (6), organic cation transporter (OCT) polymorphism (34, 47), OCT-1 inhibiting agents, age, female (48), chronic gastritis (49) and H.pylori infection (8). It is generally believed that metformin may cause diarrhea by reducing ileal bile salt reabsorption leading to elevated colonic bile salt concentration (46). Metformin can also increase the abundance of *Escherichia* and cause bloating by promoting the production of hydrogen (6). In addition to the above mechanisms, it is speculated that metformin may cause gastrointestinal adverse events by regulating SCFA-producing bacteria (50). Therefore, we designed animal experiments to confirm the relationship between metformin, SCFA-producing bacteria and gut hormones.

The food intake decreased and glucose control increased in metformin groups compared with those in the control group. In experiment A, the abundance of SCFA-producing bacteria (including *Akkemansia*, *Clostridium*, *Phascolarctobacterium*) and fecal SCFAs increased significantly in the Met+/Clin- group after treatment. The total GLP-1 level in the Met+/Clin- group was significantly higher than that in the control group. It has been reported that clindamycin induced pronounced changes in fecal SCFAs (19). In our study, the use of clindamycin reduced SCFA-producing bacteria such as *Akkemansia*, *Clostridium* and *Streptococcus*, and the levels of fecal acetic acid and propanoic acid. More interestingly, the simultaneous use of metformin and clindamycin could inhibit the promoting effect of metformin on SCFAs and GLP-1. In experiment B, the expression levels of GLP-1 in the Met+/SCFA- group and the Met-/SCFA+ group, and GLP-1, GLP-1R and PYY in the Met+/SCFA+ group increased compared with those in the control group. It has been well reported that the SCFA receptor exists on the colonic enteroendocrine L cells (51, 52). SCFAs can stimulate the secretion of both PYY and GLP-1 from wild-type primary murine colonic crypt cultures (53, 54). PYY and GLP-1 can acutely suppress appetite by inhibiting gastric emptying and reducing intestinal peristalsis (55). Therefore, we speculated that metformin can not only increase the abundance of SCFA-producing bacteria but also promote the secretion of GLP-1, therefore resulting in an increased risk of gastrointestinal adverse events and better glucose control.

There are still limitations in our study that need to be noted. This is a monocentric study, so results seem difficult to generalize. The sample size of human experimented patients is low. Moreover, this is a prospective study, and it is unable to assess a cause-effect relationship between gut microbiota and metformin-induced gastrointestinal adverse events. Although the animal experiments were grouped according to the presence or absence of Met, Clin and SCFA, the mice could not be examined that they are undergoing gastrointestinal adverse events such as abdominal pain, abdominal distention, nausea and inappetence after treatment. We can only observe that almost all mice have reduced food intake after metformin treatment. Therefore, it only speculated that metformin causes gastrointestinal adverse events may through increasing GLP-1 with the abundance of SCFA-producing bacteria.

In summary, we identified that metformin induced a significant difference in gut microbial community structure in diabetic patients. Also, there was a difference in the gut microbial community between patients with and without gastrointestinal adverse effects. We speculated that metformin promotes the secretion of intestinal hormones such as GLP-1 by increasing the abundance of SCFA-producing bacteria, which not only plays an anti-diabetic role, but also causes gastrointestinal adverse events.

## Data availability statement

The data presented in the study are deposited in the GenBank repository, accession number OP649987-OP649993.

## Ethics statement

The studies involving human participants were reviewed and approved by the Ethics Committee of Huadong Hospital Affiliated to Fudan University (Ethics Number: 2018K065). The patients/participants provided their written informed consent to participate in this study. The animal study was reviewed and approved by the Ethics Committee of Fudan University (Ethics Number: 202101004S).

## Author contributions

YH and XL: Conceptualization, methodology, writing manuscript, funding acquisition. CJ: Investigation. XT and XJ: Investigation, data curation, supervision, funding acquisition. JS: Conceptualization. ZB: Conceptualization, methodology. All authors contributed to the article and approved the submitted version.

## Funding

Natural Science Foundation of Xinjiang Uygur Autonomous Region (2021D01C025); Scientific Research Topics of Shanghai Health and Family Planning Commission (20184Y0168); Shanghai Sailing program (21YF1411700). Key Talents Program of Huadong Hospital Affiliated to Fudan University (H-1068).

## Acknowledgments

We thank Mengjuan Xue and Yixuan Qiu for animal experiments.

## Conflict of interest

The authors declare that the research was conducted in the absence of any commercial or financial relationships that could be construed as a potential conflict of interest.

## Publisher’s note

All claims expressed in this article are solely those of the authors and do not necessarily represent those of their affiliated organizations, or those of the publisher, the editors and the reviewers. Any product that may be evaluated in this article, or claim that may be made by its manufacturer, is not guaranteed or endorsed by the publisher.
